# FDG-PET/CT for lymph node staging prior to radical cystectomy

**DOI:** 10.1186/s41824-023-00170-9

**Published:** 2023-07-24

**Authors:** Vilhelm Pihl, Maria Markus, Johan Abrahamsson, Mats Bläckberg, Oskar Hagberg, Petter Kollberg, Athanasios Simoulis, Elin Trägårdh, Fredrik Liedberg

**Affiliations:** 1 Department of Urology, Skåne University Hospital, Jan Waldenströms Gata 7, 205 02 Malmö, Sweden; 2 Clinical Physiology and Nuclear Medicine, Skåne University Hospital, Malmö, Sweden; 3 Department of Translational Medicine, Lund University, Malmö, Sweden; 4 Department of Urology, Helsingborg County Hospital, Helsingborg, Sweden; 5 Department of Surgical and Perioperative Sciences, Urology and Andrology, Umeå University, Umeå, Sweden; 6 Department of Pathology, Skåne University Hospital, Malmö, Sweden

**Keywords:** Lymph node metastasis, Staging, FDG-PET/CT, Bladder cancer, Radical cystectomy, Sensitivity, Specificity

## Abstract

**Background:**

^18^F-Fluorodeoxyglucose positron emission combined with computed tomography (FDG-PET/CT) has been proposed to improve preoperative staging in patients with bladder cancer subjected to radical cystectomy (RC).

**Objective:**

Our aim was to assess the accuracy of FDG-PET/CT for lymph node staging ascertained at the multidisciplinary tumour board compared to lymph node status in the surgical lymphadenectomy specimen obtained at RC, and to explore potential factors associated with false-positive FDG-PET/CT results.

**Design, setting and participants:**

Consecutive patients with bladder cancer undergoing RC with extended lymph node dissection between 2011 and 2019 without preoperative chemotherapy in a tertial referral cystectomy unit were included in the study.

**Outcome measurements and statistical analyses:**

Sensitivity, specificity, positive and negative predictive values and likelihood ratios were calculated. Potential factors investigated for association with false-positive FDG-PET/CT were; bacteriuria within four weeks prior to FDG-PET/CT, Bacillus Calmette–Guerin (BCG) treatment within 12 months prior to FDG-PET/CT and transurethral resection of bladder tumour (TURB) within four weeks prior to FDG-PET/CT.

**Results:**

Among 157 patients included for analysis, 44 (28%) were clinically node positive according to FDG-PET/CT. The sensitivity and specificity for detection of lymph node metastasis were 50% and 84%, respectively, and the corresponding positive predictive and negative predictive values were 61% and 76%. Positive and negative likelihood ratios were 3.0 and 0.6, respectively. No association was found between bacteriuria, previous BCG treatment or TURB within 28 days and false-positive FDG-PET/CT results.

**Conclusions:**

Preoperative FDG-PET/CT prior to RC had a clinically meaningful high specificity (84%) but lower sensitivity (50%) for detection of lymph node metastases compared to lymph node status in an extended pelvic lymphadenectomy template. We could not identify any factors associated with false-positive FDG-PET/CT outcomes.

## Introduction

Preoperative staging in patients with bladder cancer subjected to radical cystectomy (RC) involves assessment of lymph nodes (LN) (European Association and of Urology Guidelines 2023). When based on computed tomography (CT) or magnetic resonance imaging (MRI) such assessment is mainly limited to measure LN size and LN morphology, with the risk of missing small metastases in lymph nodes with normal size and shape (Moschini et al. 2018). [^18^F]-fluorodeoxyglucose positron emission combined with CT (FDG-PET/CT) has been suggested to improve the accuracy in preoperative staging of LN status compared to CT (Girard et al. 2019; Vind-Kezunovic et al. 2019; Omorphos et al. 2022; Soubra et al. 2016; Bertolaso et al. 2022; Moussa et al. 2021; Einerhand et al. 2020; Crozier et al. 2019). Nonetheless, three smaller trials including 70 patients or less could not verify improved nodal staging by FDG-PET/CT (Aljabery et al. 2015; Jeong et al. 2015; Pichler et al. 2017; Cipollari et al. 2020). Hence, the objective of this study was to assess the sensitivity and specificity for FDG-PET/CT in a large and population-based cohort treated in a tertial referral centre and to explore potential factors associated with false-positive results.

## Methods

### Patients

**Fig. 1:**
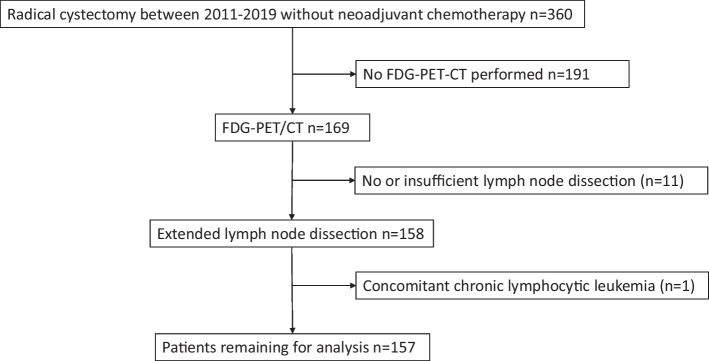
CONSORT diagram

From 2011 to 2019, 360 patients underwent RC without preoperative neoadjuvant chemotherapy (NAC) for bladder cancer in a cystectomy unit at Skåne University Hospital Malmö/Helsingborg County Hospital in Sweden. Of these, 169 patients were subjected to a preoperative FDG-PET/CT. Initially, the use of FDG-PET/CT was restricted to patients with high-risk muscle-invasive bladder cancer as a part of a prospective trial until 2014 (Kollberg et al. 2015), after which a more general use of FDG-PET/CT was applied prior to RC. Indications for RC were muscle-invasive or high-risk non-muscle-invasive bladder cancer. One patient was excluded because of concomitant chronic lymphocytic leukaemia. Another patient treated with prior radical prostatectomy and regional lymphadenectomy was excluded due to insufficient LN dissection. Ten patients where no LN dissection was performed were also excluded, and thus, 157 patients were remaining for analyses (Fig. 1). All patients were discussed at the regional multidisciplinary tumour board (MDT) at Skåne University Hospital.

### FDG-PET/CT

The Department of Medical Imaging and Physiology at Skåne University Hospital in Lund/Malmö, Kristianstad County Hospital, and Växjö County Hospital, Sweden, conducted the FDG-PET/CT scans. The systems used were Philips Gemini TF (Philips Medical Systems, Cleveland, OH), GE Discovery 690 (GE Healthcare, Milwaukee, WI, USA), GE Discovery 710 (GE Healthcare, Milwaukee, WI, USA) or GE Discovery MI (GE Healthcare, Milwaukee, WI, USA). To decrease the concentration of FDG in the urinary tract, a diuretic (intravenous furosemide 20 mg) was administrated at the same time as ^18^F-FDG (4 MBq/kg), and imaging was performed 120 min after radiopharmaceutical administration (Anjos et al. 2007). The patients were scanned from the inguinal region to the base of the skull. CT images were acquired for attenuation correction and anatomic correlation of the PET images. A diagnostic CT with intravenous and oral contrast or a low-dose CT without contrast was performed. A low-dose CT was chosen in 51/157 (32%) of the patients when a previous diagnostic CT was performed within 4 weeks of the FDG-PET/CT scan, and thus, no direct comparison between the CT and FDG-PET/CT was feasible. Instead, the outcome measure preoperative lymph node stage in the current study was derived from a reassessment and visual analyses by one nuclear medicine physician and one radiologist at the MDT prior to RC.

### Surgery

RC was performed with an extended lymph node dissection up to the aortic bifurcation, with the boundaries laterally at the genitofemoral nerve, medially the bladder wall and distally the inguinal ligament and pelvic floor. Presacral lymph nodes were also included in the lymphadenectomy specimens, which were divided in four fractions on each side (internal iliac, obturator, external iliac and common iliac). In six of the patients, dissection above the iliac bifurcation was omitted at the discretion of the operating urologist related to difficulties during dissection, locally advanced disease or intraoperative complications. All surgeries were performed as open cystectomy in one cystectomy unit (Helsingborg/Malmö) connected to the same regional MDT.

### Data analyses

The outcome measure lymph node status determined at the MDT by reassessing the FDG-PET/CT was compared to the outcome of the histopathological examination of the lymphadenectomy specimens, which were used as reference. Sensitivity, specificity, positive predictive value, negative predictive value and likelihood ratios were calculated. To investigate potential factors associated with false-positive FDG-PET/CT, comparisons were made between false-positive and true-negative results. The two groups were compared in relation to the following factors: bacteriuria within four weeks prior to FDG-PET/CT, Bacillus Calmette–Guerin (BCG) treatment within 12 months prior to FDG-PET/CT and transurethral resection of bladder tumour (TURB) within 4 weeks prior to FDG-PET/CT, with the hypothesis that inflammatory conditions in the bladder might affect the metabolism in the regional lymph nodes. Additionally, to explore the effect of size of LN metastasis in relation to false-negative outcomes, the mean size of the LN metastasis in the lymphadenectomy specimen was compared between true-positive and false-negative outcomes by comparing the proportion of lymph node metastases larger than 5 mm in the false-negative and true-positive groups. The same groups were also compared in relation to the presence of periglandular lymph node invasion.

Chi-square test was applied to compare proportions between groups. To illustrate recurrence-free survival Kaplan–Meier graphs were used using log-rank test for comparisons. The mean follow-up from date of RC was 33 (SD ± 27) months.

## Results

**Fig. 2:**
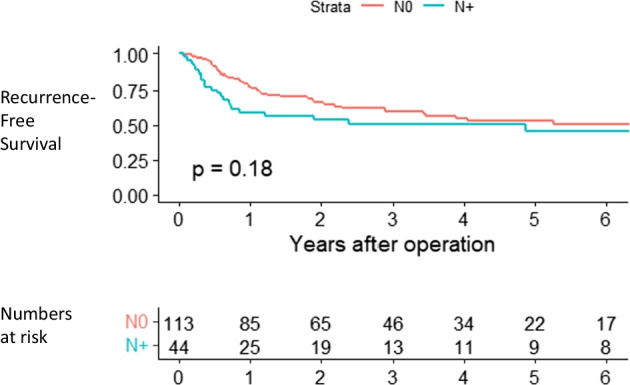
Recurrence-free survival stratified by clinical lymph node metastases

**Fig. 3:**
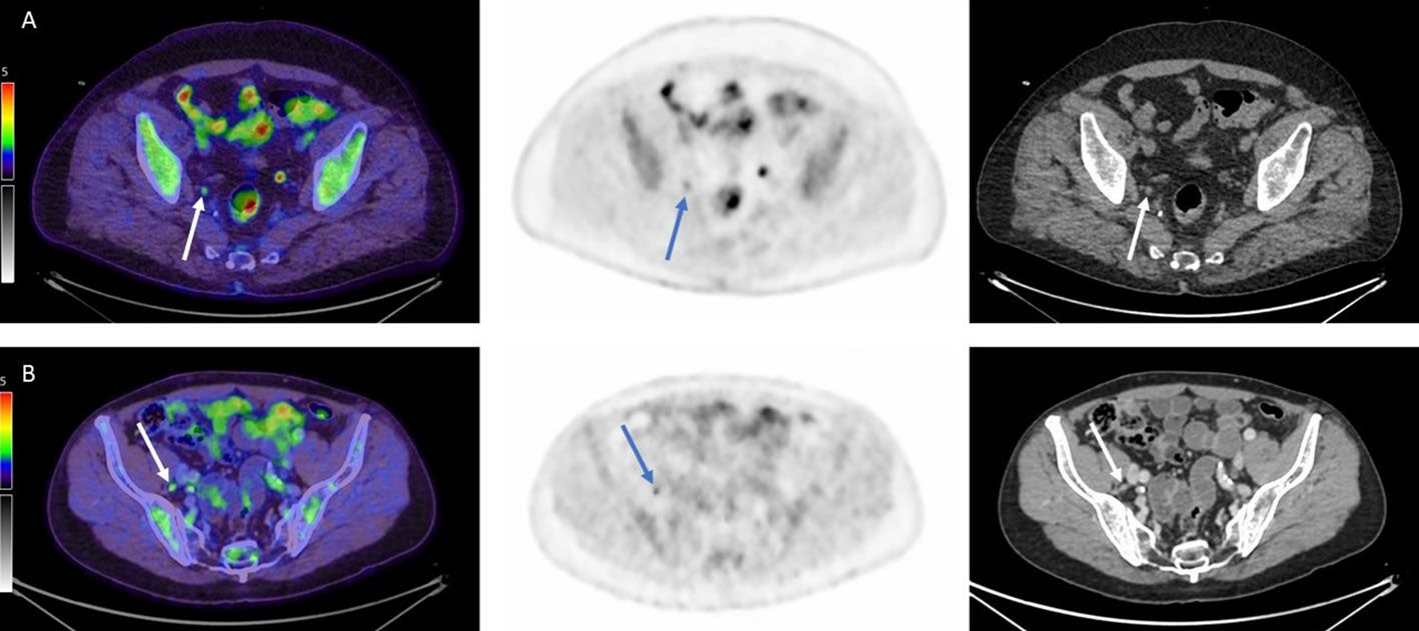
Patients with a true-positive (**A**) and false-positive (**B**) PET/CT scan, respectively. The arrows show small lymph nodes regarded as suspected lymph node metastases on PET/CT. Fused PET/CT images (left), PET images (middle) and CT images (right)

Out of the 157 patients with a mean age of 72 (SD ± 8) years, 44 (28%) were clinically node positive, according to FDG-PET/CT. Descriptive pre- and postoperative information is provided in Tables 1 and 2, respectively. Recurrence-free survival after RC stratified according to clinical lymph node status is shown in Fig. 2. The sensitivity for detection of LN metastases was 50%, i.e. of the 54 patients with lymph node metastasis in the lymphadenectomy specimen, 27 had a positive FDG-PET/CT. Corresponding to a specificity of 84%, 86 of the 103 patients without histopathological lymph node metastases had a negative FDG-PET/CT. Furthermore, the positive and negative predictive values were 61% and 76%, respectively. The positive likelihood ratio (LR +) was 3.0, and the negative likelihood ratio (LR −) was 0.6 (Table 3). Figure 3 shows one true-positive and one false-positive patient, respectively.

**Table 1:** Patient characteristics for the 157 patients

	Numbers (%)
Age mean (SD) years	72 (± 8)
*Sex (n)*	
Male	116 (74)
Female	41 (26)
Mean days (SD) from TURB to FDG-PET/CT*	34 (± 22)
Resection biopsies of the prostatic urethra/bladder neck in females	108 (69)
Mean days (SD) from FDG-PET/CT to cystectomy	48 (± 37)
Bacteriuria within 4 weeks before FDG-PET/CT	17 (11)
BCG within 12 months before FDG-PET/CT	9 (6)
*Clinical tumour stage*	
Tis	3 (2)
T1	42 (27)
T2	68 (43)
T3	28 (18)
T4	16 (10)
*Clinical node stage*	
N0	113 (72)
N1	28 (18)
N2	7 (4)
N3	9 (6)

*Only for those 143 who were examined with PET-CT after the TURB

*SD* Standard deviation

**Table 2:** Findings in the cystectomy and lymphadenectomy specimens

	Numbers (%)
*Pathological tumour stage*	
T0	17 (11)
Ta	8 (5)
Tis	16 (10)
T1	9 (6)
T2	21 (13)
T3	56 (36)
T4	30 (19)
*Pathological node stage*	
N0	103 (66)
N1	20 (13)
N2	16 (10)
N3	18 (11)
Largest node metastasis per patient (mean, SD) mm	12 (± 10)
*Periglandular invasion*	
Yes	32 (59)
No	22 (41)
Excised lymph nodes per patient (mean, SD)	36 (± 16)

**Table 3:** Diagnostic performance of FDG-PET/CT for lymph node staging

		95% CI
Sensitivity	50%	(36–64)
Specificity	84%	(75–90)
LR +	3.0	(1.8–5.0)
LR −	0.6	(0.5–0.8)
PPV	61%	(46–76)
NPV	76%	(67–84)
Accuracy	72%	(64–79)

*PPV* Positive predictive value, *NPV* Negative predictive value, *LR + and LR − * Positive and negative likelihood ratios, *CI* Confidence interval

Among the 17 false-positive cases, three patients (18%) had bacteriuria. The corresponding proportion in the true-negative group was 10%, as 9 patients of the 86 true-negative cases had bacteriuria (NS). None of the false-positive cases were treated with BCG within 12 months prior to FDG/PET-CT. Nine (53%) of the false-positive cases underwent TURB within 28 days before FDG-PET/CT. In the true-negative cases group, the corresponding rate was 37% (32/86) (NS). The mean number of days from TURB to PET in the false-positive group was 31 (SD ± 16) compared to 37 (SD ± 23) days in the true-negative group.

In the group of true-positive FDG-PET/CT-investigations, the largest lymph node metastasis found in the lymphadenectomy specimens was > 5 mm in 25 out of 27 (93%) patients. In the false-negative group, the corresponding proportion was 21/27 (78%) (NS). Correspondingly, the mean size of the largest lymph node metastasis in the true-positive group was 16 mm (SD ± 13) and in the false-negative group 9 mm (SD ± 6). Of the 54 patients with lymph node metastases, 32 had periglandular tumour invasion, 17 out of 27 (63%) in the true-positive group and 15 out of 27 (56%) in the false-negative group (NS).

Of the 157 patients, 85 were scanned on a Philips Gemini TF PET/CT, 41 on a Discovery MI PET/CT and the remaining patients were scanned on a Discovery 690 PET/CT and 710 PET/CT, respectively. A separate analysis of patients scanned on the newest PET/CT system (Discovery MI) resulted in a sensitivity of 56%, a specificity of 81%, a PPV of 45%, a NPV of 87%, a LR + of 3.0 and a LR −  of 0.6. For the oldest PET/CT system (Philips Gemini TF), the sensitivity was 45%, specificity 85%, PPV 65%, NPV 71%, LR + 3.0 and LR −  0.6. Due to a small number of patients, separate analyses were not performed for the Discovery 690 or Discovery 710 systems.

## Discussion

The sensitivity and specificity for lymph node staging by FDG-PET/CT reassessed at MDT prior to RC compared to an extended lymph node dissection template up to the aortic bifurcation was 50% and 84%, respectively. The reported specificity of FDG-PET/CT in two recent reviews and meta-analyses was higher (92%) (Crozier et al. 2019; Ha et al. 2018), as was the sensitivity (56% and 57%, respectively). The lower sensitivity found in the present series might partly be related to that the comparator was an extended lymphadenectomy with mean 36 nodes excised, with a high probability of identifying all lymph node-positive patients. Furthermore, a lower specificity in the current series might be related to selection mechanisms. Patients with more clear or extensive lymph node spread according to FDG-PET/CT were more likely to be selected for induction chemotherapy, with FDG-PET/CT as a measure of chemotherapy response (Abrahamsson et al. 2022). With these patients excluded from the current cohort, the specificity might have been reduced by an accumulation of a more ambiguously FDG-PET/CT-positive patients compared to trials where neoadjuvant chemotherapy was not an exclusion criteria (Crozier et al. 2019; Ha et al. 2018). Additionally, the current study evaluated the FDG-PET/CT examinations by visual analysis only (and then dichotomized into positive or negative) and not by maximum standardized uptake values (SUV_max_) that with increased values has been correlated with increased specificity (Vind-Kezunovic et al. 2019).

The mean age of 72 years in the present study was higher than in all but one of the cohorts included in two recent systematic reviews reporting sensitivity and specificity for FDG-PET/CT in invasive bladder cancer (Crozier et al. 2019; Ha et al. 2018). This is also likely related to selection mechanisms since the majority of patients with muscle-invasive bladder cancer below 76 years of age in our practice were treated with neoadjuvant chemotherapy prior to RC [91% during 2019 (Swedish National Registry of Urinary Bladder Cancer 2023)]. As such neoadjuvant chemotherapy was an exclusion criterium to enable accurate assessment of lymph node metastases in the lymphadenectomy specimen without preoperative chemotherapy causing ypN0, the remaining patients ineligible for cisplatin-based neoadjuvant chemotherapy were older.

None of factors investigated (bacteriuria, prior BCG treatment and FDG-PET/CT within 28 days of TURB) were associated with false-positive FDG-PET/CT outcomes, although the power to detect differences was limited related to few observations. To our knowledge, no such associations have been reported previously in the literature.

The recurrence-free survival curves for patients with node-positive and node-negative disease did not separate significantly in the current study. Similar lack of separating survival curves was reported when stratifying patients above and below SUV_max_ values of 2, but a significant difference was found when using SUV_max_ values above and below 4, in a study excluding patients who had neoadjuvant chemotherapy (Vind-Kezunovic et al. 2019). Still, other putative benefits by performing a FDG-PET/CT prior to RC could be that surgical lymphadenectomy can be tailored according to FDG-PET/CT findings, the possibility of response-evaluating induction chemotherapy by FDG-PET/CT in node-positive disease allowing for earlier switch to second-line treatment options (Abrahamsson et al. 2022), detection of distant metastases and secondary primary malignancies and increased staging accuracy in individuals with contraindications to iodine contrast. Based on those different scenarios, the composite outcome altered treatment based on FDG-PET/CT findings prior to RC has been reported in one out of five patients (Voskuilen et al. 2022). Still, there are discrepancies in how different guidelines interpret the current evidence, resulting in different recommendations regarding the use of preoperative FDG-PET/CT (Omorphos et al. 2022).

The major limitation of the current study in relation to the primary outcome accuracy of FDG-PET/CT for lymph node staging is the exclusion of patients with muscle-invasive bladder cancer receiving either neoadjuvant or induction chemotherapy in case of regional lymph node metastasis. The lack of possibilities to perform a direct comparison between lymph node status by CT and FDG-PET/CT as 51 patients were investigated with a low-dose CT only is also a study limitation. Also, the use of four different generations of PET-systems seems to slightly affect the results, although the small numbers make this finding uncertain. Still, the high specificity (84%) reported for lymph node metastases in the present study can be applied in clinical care to recommend patients preoperative induction chemotherapy in the wake of a new adjuvant treatment option with nivolumab, where an especially large effect size has been reported in subgroup analyses for patients treated with preoperative chemotherapy (Bajorin et al. 2021). Consequently, preoperative FDG-PET/CT outcomes are allowing for sequencing and maximizing perioperative systemic treatments in conjunction with radical surgery for those patients having the worst prognosis.

## Conclusions

The sensitivity and specificity for lymph node metastases compared to an extended lymph node dissection were 50% and 84% in the current study, respectively. The high specificity can be utilized to escalate treatment intensity by adding induction chemotherapy prior to RC.

## Data Availability

The datasets can be made available from the corresponding author on reasonable request.

## Abbreviations

BCG: Bacillus Calmette–Guerin
CT: Computed tomography
CI: Confidence interval
FDG-PET: ^18^F-Fluorodeoxyglucose positron emission
LR +: Positive likelihood ratio
LR −: Negative likelihood ratio
LN: Lymph nodes
MRI: Magnetic resonance imaging
MDT: Multidisciplinary tumour board
NAC: Neoadjuvant chemotherapy
NPV: Negative predictive value
PPV: Positive predictive value
RC: Radical cystectomy
SD: Standard deviation
SUV_max_: Maximum standardized uptake values
TURB: Transurethral resection of bladder tumour

## References

[CR1] Abrahamsson J, Kollberg P, Almquist H (2022). Complete metabolic response with [^18^ F]fluorodeoxyglucose-positron emission tomography/computed tomography predicts survival following induction chemotherapy and radical cystectomy in clinically lymph node positive bladder cancer. BJU Int.

[CR2] Aljabery F, Lindblom G, Skoog S (2015). PET/CT versus conventional CT for detection of lymph node metastases in patients with locally advanced bladder cancer. BMC Urol.

[CR3] Anjos DA, Etchebehere EC, Ramos CD (2007). 18F-FDG PET/CT delayed images after diuretic for restaging invasive bladder cancer. J Nucl Med.

[CR4] Bajorin DF, Witjes JA, Gschwend JE (2021). Adjuvant Nivolumab versus Placebo in muscle-invasive urothelial carcinoma. N Engl J Med.

[CR5] Bertolaso P, Brouste V, Cazeau AL (2022). Impact of ^18^ FDG- PET CT in the management of muscle invasive bladder cancer. Clin Genitourin Cancer.

[CR6] Cipollari S, Carnicelli G, Bicchetti M (2020). Utilization of imaging for staging in bladder cancer: is there a role for MRI or PET-computed tomography?. Curr Opin Urol.

[CR7] Crozier J, Papa N, Perera M (2019). Comparative sensitivity and specificity of imaging modalities in staging bladder cancer prior to radical cystectomy: a systematic review and meta-analysis. World J Urol.

[CR8] Einerhand SMH, van Gennep EJ, Mertens LS (2020). 18F-fluoro-2-deoxy-D-glucose positron emission tomography/computed tomography in muscle-invasive bladder cancer. Curr Opin Urol.

[CR9] European Association of Urology Guidelines, Accessed 10 Apr 2023 from https://uroweb.org/guidelines/muscle-invasive-and-metastatic-bladder-cancer/chapter/diagnostic-evaluation

[CR10] Girard A, Rouanne M, Taconet S (2019). Integrated analysis of ^18^F-FDG PET/CT improves preoperative lymph node staging for patients with invasive bladder cancer. Eur Radiol.

[CR11] Ha HK, Koo PJ, Kim SJ (2018). Diagnostic accuracy of F-18 FDG PET/CT for preoperative lymph node staging in newly diagnosed bladder cancer patients: a systematic review and meta-analysis. Oncology.

[CR12] Jeong IG, Hong S, You D (2015). FDG PET-CT for lymph node staging of bladder cancer: a prospective study of patients with extended pelvic lymphadenectomy. Ann Surg Oncol.

[CR13] Kollberg P, Almquist H, Bläckberg M (2015). [(18)F]Fluorodeoxyglucose - positron emission tomography/computed tomography improves staging in patients with high-risk muscle-invasive bladder cancer scheduled for radical cystectomy. Scand J Urol.

[CR14] Moschini M, Morlacco A, Briganti A (2018). Clinical lymphadenopathy in urothelial cancer: a transatlantic collaboration on performance of cross-sectional imaging and oncologic outcomes in patients treated with radical cystectomy without neoadjuvant chemotherapy. Eur Urol Focus.

[CR15] Moussa M, Chakra MA, Saad W (2021). The role of 18F-FDG PET/CT scan compared to CT-scan alone for lymph node staging before radical cystectomy in patients with bladder cancer. Urol Oncol.

[CR16] Omorphos NP, Ghose A, Hayes JDB (2022). The increasing indications of FDG-PET/CT in the staging and management of invasive bladder cancer. Urol Oncol.

[CR17] Pichler R, De Zordo T, Fritz J (2017). Pelvic lymph node staging by combined ^18^F-FDG-PET/CT imaging in bladder cancer prior to radical cystectomy. Clin Genitourin Cancer.

[CR18] Soubra A, Hayward D, Dahm P (2016). The diagnostic accuracy of 18F-fluorodeoxyglucose positron emission tomography and computed tomography in staging bladder cancer: a single-institution study and a systematic review with meta-analysis. World J Urol.

[CR19] Swedish National Registry of Urinary Bladder Cancer, Accessed 10 Apr 2023 from https://statistik.incanet.se/Urinblasecancer/

[CR20] Vind-Kezunovic S, Bouchelouche K, Ipsen P (2019). Detection of lymph node metastasis in patients with bladder cancer using maximum standardised uptake value and ^18^F-fluorodeoxyglucose positron emission tomography/computed tomography: results from a high-volume centre including long-term follow-up. Eur Urol Focus.

[CR21] Voskuilen CS, van Gennep EJ, Einerhand SMH (2022). Staging ^18^F-fluorodeoxyglucose positron emission tomography/computed tomography changes treatment recommendation in invasive bladder cancer. Eur Urol Oncol.

